# LGBT+ Older Adults Report Higher Rates of Loneliness and Other Adverse Social Determinants of Health

**DOI:** 10.1093/geroni/igaf122.004

**Published:** 2025-12-31

**Authors:** Jace Flatt, Joel Anderson, Ethan Cicero, Jalal Uddin, Erin Rook

**Affiliations:** University of Nevada Las Vegas, Nevada, United States; University of Tennessee, Knoxville, Tennessee, United States; Emory University, Atlanta, Georgia, United States; University of Nevada Las Vegas, Nevada, United States; University of Nevada Las Vegas, Nevada, United States

## Abstract

While loneliness tends to decline with age, lesbian, gay, bisexual, transgender, and additional identity (LGBT+) older adults face heightened risks of loneliness and adverse social determinants of health (SDoH). We examined data from 33 U.S. states and 2 territories from the 2023 Behavioral Risk Factor Surveillance System that included optional SDoH and sexual orientation and gender identity (SOGI) modules. The sample included adults aged 65+ (mean:73.5 years, range:65-80+) who identified as a LGBT+ (n = 2356) and non-LGBT + (n = 68,546). Loneliness was defined as feeling socially isolated from others always/usually/sometimes. Multivariable logistic regression models examined associations between loneliness and LGBT+ identity adjusting for demographic characteristics (age, race/ethnicity, education and income). Compared with non-LGBT+ older adults, LGBT+ older adults were slightly younger and unmarried/not partnered (61% vs. 46%), and reported annual incomes ≤$20,000 (12% vs. 8%), poor/fair health (25% vs. 22%), greater depression (22% vs. 15%), and greater functional impairment (10% vs. 9%). In terms of SDoH, LGBT+ older adults also reported greater food insecurity (11% vs. 7.0%) and housing insecurity (5% vs. 3%) compared with non-LGBT+ older adults. Nearly one third of LGBT+ older adults reported current loneliness compared with one fifth of non-LGBT+ older adults (p < 0.001). After accounting for demographics, LGBT+ older adults were 30% more likely to report loneliness (aOR:1.30; 95% CI:1.17, 1.44) than non-LGBT+ older adults. This study highlights higher rates of loneliness among LGBT+ older adults compared with non-LGBTQ+ older adults and the critical need for interventions to bolster social connectedness and support for LGBT+ older adults.

